# Fano resonance based defected 1D phononic crystal for highly sensitive gas sensing applications

**DOI:** 10.1038/s41598-020-75076-8

**Published:** 2020-10-21

**Authors:** Shrouk E. Zaki, Ahmed Mehaney, Hekmat M. Hassanein, Arafa H. Aly

**Affiliations:** 1 Theoretical Physics Department, National Research Centre, Dokki, Cairo, Egypt; 2 TH-PPM Group, Physics Department, Faculty of Sciences, Beni-Suef University, Beni-Suef, Egypt

**Keywords:** Mathematics and computing, Physics

## Abstract

The defected acoustic band gap materials are promising a new generation of sensing technology based on layered cavities. We introduced a novel 1D defected phononic crystal (1D-DPC) as a high-sensitive gas sensor based on the Fano resonance transmitted window. Our designed (Lead–Epoxy) 1D-DPC multilayer has filled with a defect layer with different gases at different temperatures. In this study, Fano resonance—based acoustic band gap engineering has used to detect several gases such as O_2_, CO_2_, NH_3_, and CH_4_. For the first time, Fano resonance peaks appeared in the proposed gas sensor structures which attributed to high sensitivity, Q-factor, and figure-of-merit values for all gases. Also, the relation between the Fano resonance frequency and acoustic properties of gases at different temperatures has been studied in detail. The effect of the damping rate on the sensitivity of the gas sensor shows a linear behavior for CO_2_, O_2_, and NH_3_. Further, we introduced the effect of temperature on the damping rate of the incident waves inside the 1D-DPC gas sensor. The highest sensitivity and figure of merit were obtained for O_2_ of 292 MHz/(kg/m^3^) and 647 m^3^/Kg, respectively. While the highest figure-of-merit value of 60 °C^−1^ at 30 °C was attributed to O_2_. The transfer matrix method is used for calculating the transmission coefficient of the incident acoustic wave. We believe that the proposed sensor can be experimentally implemented.

## Introduction

Detection of harmful gases, especially toxic and explosive gases in ambient conditions has a significant role in both urban and industrial environments^1^. Gas sensing technology of detecting gases such as CO_2_, CO, CH_4_, and NO_x_ is considered a vital process to prevent harm to human life. Gas sensing can be used for a wide range of applications including the medical sector, environmental monitoring, and manufacturing control^2^. For instance, controlling respiratory gases in hospitals is being a matter of life or death^3,4^. Greenhouse gases such as CO_2_ and CH_4_ are considered as very harmful gases, as they have the property of absorbing infrared radiation (IR) emitted from Earth's surface and reradiating it back to Earth's surface, thus trapping the heat in the Earth's atmosphere^5,6^. While O_2_ is considered a strong oxidizer and vigorously accelerate combustion gas^5^. Gas sensors have many different working principles based on different methods such as phononic crystal (PC)^1^, semiconductor thin films^6–12^, catalytic^13^, and optical gas sensors^14^. Among them, the PC-based systems are perfect candidates to develop ultrasonic gas sensors^1^, as it is well known that PCs are considered as the most dispersive materials for acoustics—elastic waves. Moreover, the acoustic PC gas sensor is considered as a hot research topic and promising for achieving practical and low-cost sensing^1^. PC is a smart periodic structure of solid materials and/or fluids with different acoustic wave velocities^15^. The unique property of PC is that it exhibits frequency stop and pass bands, these bands caused by the multiple scattering of waves within a PC.

Recently, using PC as a gas and liquid sensor has attracted significant attention due to its novel applications in sensing^16–18^, wave-guiding^19,20^, acoustic focusing^21^, lensing^22,23^ and topological phononics^24,25^. Nevertheless, more advantages of using PC devices is the ability to effect on the reference and the target gas by modulating any external influence such as pressure, or temperature^26^. On the other hand, the DPC with different gases embedded in the solid matrix has exceptional advantages over other ordinary PC^27^. For instance, by creating defects and breaking the PC periodicity, resonant modes can be generated inside the phononic band gaps, which increase the novelty of such periodic structures more than the perfect ones^28^. Since the appearance of the celebrated Fano resonance more than 50 years ago^29^, Fano resonance attracts great attention due to their featuring sharp asymmetric line shape, originating from destructive interference between narrow discrete state and broad continuum state^30,31^. It has great attention due to its potential and wide applications in sensors and optical switches^32^. The Fano resonance phenomenon has been studied in PC and other periodic structures and it has been used in the acoustic waveguide techniques of PC structures^33^. Limonov et al., studied theoretically and experimentally the Fano resonance, induced transparency, Kerker and Borrmann effects, and parity-time symmetry breaking in photonic crystal design^34^. In addition, it introduced in another applications such as PC resonators^35^, optomechanical devices^36^, acoustic-wave guiding^37^, and PC radiation detectors^38^. On the other hand, the acoustic Fano-resonance based 1D-DPC gas sensor system, in which acoustic frequencies have sharp resonance transmission peaks with high sensitivity and quality factors did not cover before. It is for the first time to show that the Fano resonance that appeared in our designed 1D-PC gas sensor plays dramatic enhancements in the gas sensor performance. Moreover, the Fano resonance phenomena don’t appear in all previous work of 1D or 2D phon PCs gas sensors as shown in the previous work^1,39–42^.

In the previous studies, Cicek et al. experimentally designed a 2D surface PC for an ultrasonic gas sensing^1^. Also, Cicek et al. experimentally and theoretically demonstrated an ultrasonic gas sensor based on the evanescent coupling of spoof surface acoustic waves between two surface PCs towards CO_2_^39^. In addition, Cheeke et al., represented the interaction between the acoustic wave and gaseous species in details^40^. Further, Kaya et al., represented numerically and experimentally a gas sensor based on a 1D-PC to measure CO_2_ level in air^41^. Furthermore, Mehaney et al. theoretically demonstrated a CO_2_ gas sensor based on a 1D porous silicon PC sandwiched between two thin rubber layers to measure CO_2_ pollutions in the surrounding air^42^. Moreover, the PC plays a significant role in the field of smart sensors such as liquid sensor^43–47^, acoustic biosensor^48,49^, chemical sensor^43,44^, bio-chemical sensor^50^, acoustic wave-guide^33^, and biodiesel sensor^51,52^ have been introduced before.

However, the field of studying 1D-DPC as a toxic gas sensor based on the transfer matrix method (TMM) and Fano resonance still not covered yet, which is the main focus of this study. Our theoretical study based on 1D-PC as a gas sensor covered many factors that still not covered in the previous studies. Moreover, our proposed 1D-DPC gas sensor can easily fabricate experimentally and theoretically due to the use of 1D multilayered structures in sensing applications as introduced before^26,43,52–55^. Also, our gas sensor can work under hard conditions such as high temperature and pressure due to the presence of cheap materials such as lead and epoxy. In addition, it doesn’t need complex electric components to work. Further, we can use it at any place as the acoustic sensors can be easily affected by any change in the environment.

Firstly, our proposed study covered the detection of several nontoxic and toxic gases such as O_2_, CO_2_, NH_3_, and CH_4_. Secondly, it is the first time to observe the Fano resonance peaks in 1D-DPC gas sensor with their impressive contributions to the sensor performance. These Fano resonance peaks attributed to high sensitively records, Q-factor, and figure-of-merit values for all proposed gases. Thirdly, the role of the damping rate factor at room temperature and high temperature has shown a means to our gas sensor design structure for practical applications. Moreover, the effect of temperature on the Fano resonance frequency towards the gases and performance of our gas sensor has been studied in detail.

### 1D-DPC gas sensing design

**Figure 1:**
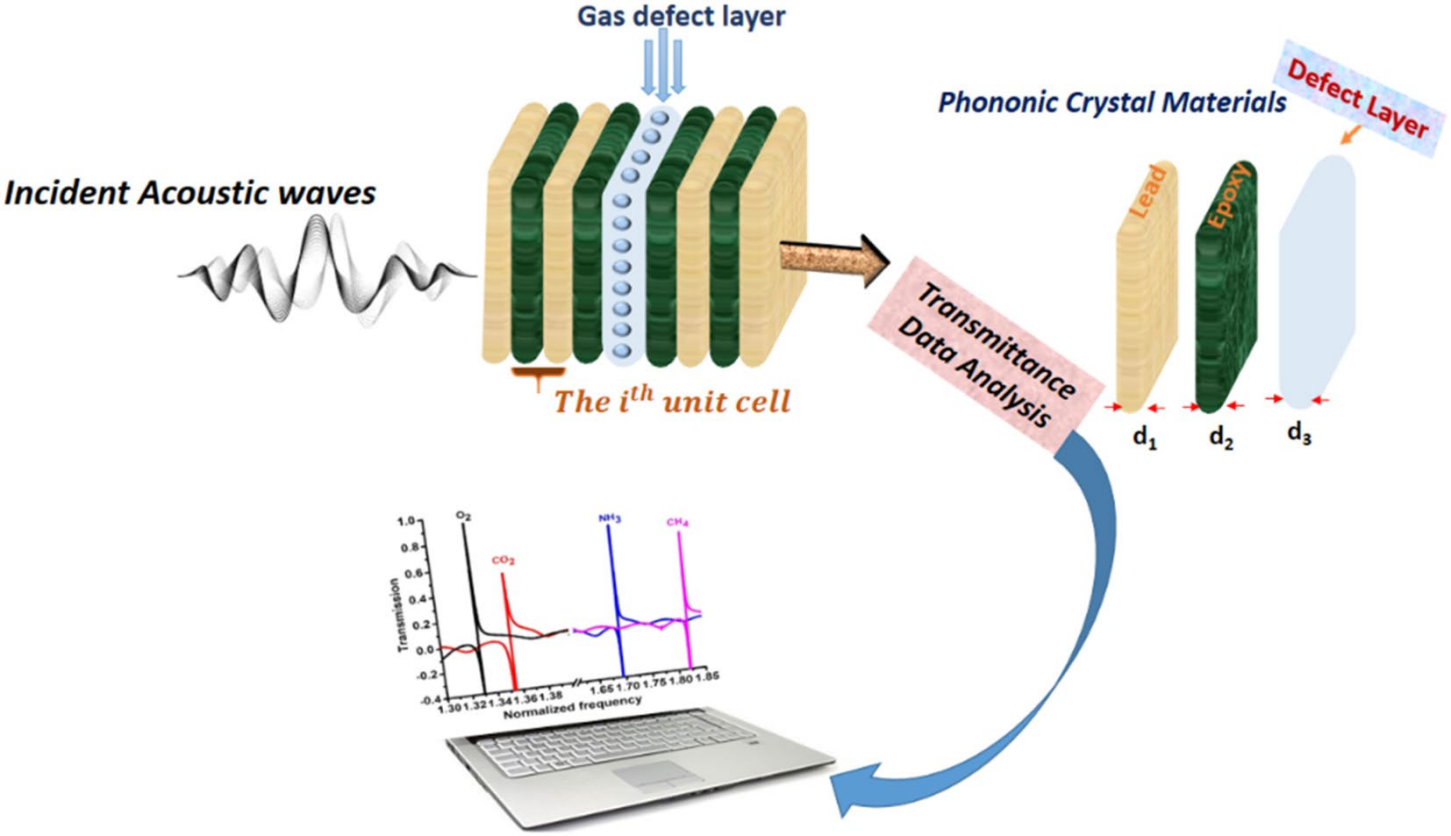
A schematic diagram of a 1D-DPC gas sensor structure consists of a periodic multilayer of lead and epoxy with a defect layer filled with different gases.

Our designed gas sensor is considered as a defective binary structure composed of two different layers of (Lead–Epoxy) repeated in N = 4 unit cells with equal thickness, followed by insertion of a defect layer in the middle of the structure [(Lead/Epoxy)^2^ − (sensing material) − (Lead/Epoxy)^2^]. The defect layer was filled with different gases that have been tested separately. Firstly, we filled the defect layer with air as a reference for all the target gases and we considered the air resonance frequency as the reference for the position of resonance peaks towards the different gases. We have considered the lead and epoxy materials due to the large acoustic impedance mismatching factors. The key to be taken as input parameter and shows its gas sensor attempt is the mechanical properties of the designed layers and the gas-filled the defect layer. These mechanical properties are represented by the sound speed and mass density. A schematic diagram of the 1D-DPC gas sensor structure is shown in Fig. 1. For each layer that used in our gas sensor, the density, longitudinal sound velocity, and physical thicknesses are summarized in Table 1.

**Table 1:** Gives the values of the acoustic parameters of each layer used in this study.

Materials	Density (kg/m^3^)	Longitudinal sound speed ${C}_{L}$ (m/s)	Thickness (μm)
Lead	10,760	1960	1
Epoxy	1140	2770	1
CO_2_	1.8393	267	1.5
NH_3_	0.7069	430	1.5
O_2_	1.314	326	1.5
CH_4_	0.659	445	1.5
Air (reference)	1.2047	343	1.5

### Theoretical model

The presented configuration of our gas sensor is a binary structure as shown in Fig. 1. Nowadays, the periodic binary 1D-DPC structures have attracted significant attention due to their high sensing performance compared with the perfect PC systems^18,45^. There are several techniques have been developed to investigate the propagation of acoustics waves through the PC including the TMM, plane wave expansion(PWE) method, the finite difference time domain (FDTD) method^56–58^. However, of these techniques, only the TMM method is a suitable method for calculating the transmission of incident acoustic waves through the multilayer structure^27,45,52^.

When the acoustic wave incident normally on our proposed 1D-DPC gas sensor, the mechanical properties of the layers such as sound speed and density are changing periodically, and the incident acoustic wave disperses into several waves inside the structure.

For an acoustic wave incident normally on the 1D PC structure from the left to the right as shown in Fig. 1, the equation of motion in each layer can be written in the following form^59,60^:

1 $$\frac{\partial \sigma }{\partial \mathrm{x}}+f=\rho \frac{{\partial }^{2}\mathrm{u}}{\partial {t}^{2}}$$

where σ = σ(x, t), f = f(x, t), ρ = ρ(x, t), and u = u(x, t) denote the stress, external body force, material density, and displacement, respectively. In Eq. (1). The stress of elastic material for which is given by:

2 $$\sigma = E\frac{\partial u}{\partial \mathrm{x}}$$

where E = E(x) is Young’s modulus of the material. By substituting Eq. (2) into Eq. (1):

3 $$E\frac{{\partial }^{2}u}{\partial {x}^{2}}= \rho \frac{{\partial }^{2}u}{\partial {t}^{2}}$$

Using Eq. (3) to study the acoustic wave propagation through the 1D PC multilayer structure as follows:

4 $$ u\left( {{\text{x}},{\text{ t}}} \right) = Ae^{i(Kx - \omega t)} $$

where ($A$) is the wave amplitude, $K$ is the wavenumber, ω is angular frequency. By Substituting Eq. (4) into Eq. (3) provides the wave equation for a gas or liquid as follows:

5 $$\frac{{\partial }^{2}u}{\partial {x}^{2}}= \frac{1}{{C}_{L}^{2}}\frac{{\partial }^{2}u}{\partial {t}^{2}}$$

Consider a 1D PC consists of N unit cells, each cell has two layers and denoted by the subscript *j* = 1, 2 for a layer *j* in the unit cell, the associated material properties, which are constant, are The thickness, density, Young’s modulus, cell length, Lame’ constant, shearing modulus are given as ${d}_{j}$, ${\rho }_{j}$, ${E}_{j,}$ $\mathrm{a}={\sum }_{\mathrm{j}=1}^{\mathrm{n}}{d}_{j}$, ${\lambda }_{j},$ ${\mu }_{j}$. The subscript j = 1, 2, and 3 represent the layer type. The acoustic wave velocity in each layer is ${\mathrm{C}}_{\mathrm{L}}$ = $\sqrt{\mathrm{E}/\uprho }$. The solution to Eq. (5) can be expressed in the form:

6 $$  u\left( {{\text{x}},{\text{t}}} \right) = \left[ {A_{ + }^{{(j)}} e^{{iK_{j} X}}  + A_{ - }^{{(j)}} e^{{ - iK_{j} X}} } \right]e^{{ - i\omega t}}   $$

where ${K}_{j}$ = $\frac{2\pi f}{{\mathrm{C}}_{\mathrm{L}}j}$ is the wavenumber, $f$ is the frequency of the incident wave, the coefficients are* A*_+_* and  A*_−_ are the amplitudes of transmitted and reflected waves, respectively.

The formula of the incident wave equation through the gas layer is expressed as in Eq. (7). Concerning the interaction between gases and PC, we supposed to insert a defect layer inside the perfect phononic structure. It well known that the acoustic waves is considered as a one kind of pressure fluctuation that can exist in any compressible gas. Through the gas medium the neutral atoms and molecules can conduct the transmission of sound waves through it and the equation that describe the acoustic wave propagation in gases defined by the formula Eq. (7)^61^:

7 $$ \begin{aligned} & \frac{{\partial^{2} p}}{{\partial x^{2} }} - \frac{\rho }{\beta }\frac{{\partial^{2} p}}{{\partial t^{2} }} = 0 \\ & \frac{{\partial^{2} p}}{{\partial x^{2} }} - \frac{1}{{C_{0}^{2} }}\frac{{\partial^{2} p}}{{\partial t^{2} }} = 0 \\ \end{aligned} $$

where ${C}_{0}=\sqrt{\frac{\beta }{\rho }}$ the speed of sound in gas layer, $p$ is the instantaneous pressure in the gas, and ρ is the mass density and β is the bulk modulus. The solution of Eq. (7) can be given by Eq. (8):

8 $$  p\left( {{\text{x}},{\text{t}}} \right) = \left[ {P_{ + }^{{(j)}} e^{{iK_{j} X}}  + P_{ - }^{{(j)}} e^{{ - iK_{j} X}} } \right]e^{{i\omega t}}   $$

When a sound wave travels through gas medium, the local pressure fluctuations and local density fluctuations are generated. The acoustic impedance of gas is related to the speed of sound in it through Eq. (9)^62^:

9 $$Z=\rho \times {C}_{0}$$

where ρ is the gas density and ${C}_{0}$ is the speed of sound in gas. Thus, the acoustic impedance that is acting in opposition to the wave propagation increases with an increase in medium density as well as an increase in the speed of sound.

There are two conditions that must be satisfied at the interfaces between layers: the continuity of displacement and the continuity of stress. We can provide the stress equation by substituting Eq. (6) into Eq. (2) as shown:

10 $$  \begin{aligned}    & \sigma \left( x \right) = EiK_{j} \left[ {A_{ + }^{{\left( j \right)}} e^{{iK_{j} X}}  - A_{ - }^{{\left( j \right)}} e^{{ - iK_{j} X}} } \right] \\     & \sigma \left( x \right) = iZ_{j} [A_{ + }^{{\left( j \right)}} e^{{iK_{j} X}}  - A_{ - }^{{\left( j \right)}} e^{{ - iK_{j} X}}  \\  \end{aligned}   $$

where ${Z}_{j}={E}_{j}{K}_{j}$ . So we can write the spatial components of Eq. (10) in the form as.

11 $$ \left[ {\begin{array}{*{20}c} {{\text{u}}\left( {\text{x}} \right)} \\ {\sigma \left( {\text{x}} \right)} \\ \end{array} } \right] = \left[ {\begin{array}{*{20}c} 1 & 1 \\ {iZ_{j} } & { - iZ_{j} } \\ \end{array} } \right]\left[ {\begin{array}{*{20}c} {A_{ + }^{\left( j \right)} e^{{iK_{j} X}} } \\ {A_{ - }^{\left( j \right)} e^{{ - iK_{j} X}} } \\ \end{array} } \right] = B_{j} \left[ {\begin{array}{*{20}c} {A_{ + }^{\left( j \right)} e^{{iK_{j} X}} } \\ {A_{ - }^{\left( j \right)} e^{{ - iK_{j} X}} } \\ \end{array} } \right] $$

where ${B}_{j}$ is the wave matrix at the interface between the two layers. This allows us to substitute the relation ${X}_{R}^{j}$= ${X}_{L}^{j}+ {d}_{j}$ where ${X}_{R}^{j}$ and ${X}_{L}^{j}$ denote the position of the right and left boundary, respectively, of layer *j* thus relate the displacement and stress at ${X}_{L}^{j}$ to those at ${X}_{R}^{j}$.

12 $$ \left[ {\begin{array}{*{20}c} {{\text{u}}\left( {X_{R}^{j} } \right)} \\ {\sigma \left( {X_{R}^{j} } \right)} \\ \end{array} } \right] = B_{j} \left[ {\begin{array}{*{20}c} {e^{{iK_{j} d_{j} }} } & 0 \\ 0 & {e^{{ - iK_{j} d_{j} }} } \\ \end{array} } \right]\left[ {\begin{array}{*{20}c} {A_{ + }^{\left( j \right)} e^{{iK_{j} X_{L}^{j} { }}} } \\ {A_{ - }^{\left( j \right)} e^{{ - iK_{j} X_{L}^{j} }} } \\ \end{array} } \right] = B_{j} D_{j} \left[ {\begin{array}{*{20}c} {A_{ + }^{\left( j \right)} e^{{iK_{j} X_{L}^{j} { }}} } \\ {A_{ - }^{\left( j \right)} e^{{ - iK_{j} X_{L}^{j} }} } \\ \end{array} } \right] $$

where ${D}_{j}$ = $$\left[\begin{array}{cc}{e}^{{iK}_{j}{d}_{j}}& 0\\ 0& {e}^{{-iK}_{j}{d}_{j}}\end{array}\right]$$ is wave matrix through each layer.

$$ {\text{And,}}\quad \left[ {\begin{array}{*{20}c} {{\text{u}}\left( {X_{L}^{j} } \right)} \\ {\sigma \left( {X_{L}^{j} } \right)} \\ \end{array} } \right] = B_{j} \left[ {\begin{array}{*{20}c} {A_{ + }^{\left( j \right)} e^{{iK_{j} X_{L}^{j} { }}} } \\ {A_{ - }^{\left( j \right)} e^{{ - iK_{j} X_{L}^{j} }} } \\ \end{array} } \right] $$

We can rewrite Eq. (12) as:

13 $$ \left[ {\begin{array}{*{20}c} {{\text{u}}\left( {X_{R}^{j} } \right)} \\ {\sigma \left( {X_{R}^{j} } \right)} \\ \end{array} } \right] = B_{j} D_{j} \left[ {\begin{array}{*{20}c} {A_{ + }^{\left( j \right)} e^{{iK_{j} X_{L}^{j} { }}} } \\ {A_{ - }^{\left( j \right)} e^{{ - iK_{j} X_{L}^{j} }} } \\ \end{array} } \right] = B_{j} D_{j} B_{j}^{ - 1} \left[ {\begin{array}{*{20}c} {u\left( {X_{L}^{j} } \right)} \\ {\sigma \left( {X_{L}^{j} } \right)} \\ \end{array} } \right] = T_{j} \left[ {\begin{array}{*{20}c} {u\left( {X_{L}^{j} } \right)} \\ {\sigma \left( {X_{L}^{j} } \right)} \\ \end{array} } \right] $$

Equation (13) relates the displacement and stress at ${X}_{L}^{j}$ to those at ${X}_{R}^{j}$ of the same layer *j, and* ${T}_{j}$, is the transfer matrix for layer j, has the expanded form^60^:

14 $$ T_{j} = \left[ {\begin{array}{*{20}c} {\cos \left( {K_{j} d_{j} } \right)} & {\frac{1}{{Z_{j} }}\sin \left( {K_{j} d_{j} } \right)} \\ { - Z_{j} \sin \left( {K_{j} d_{j} } \right)} & {\cos \left( {K_{j} d_{j} } \right)} \\ \end{array} } \right] $$

Since the transfer matrix is valid for any layer and ${X}_{L}^{j} \equiv {X}_{R}^{(j-1)}$, the result in Eq. (14) can be extended across several layers, so we can write the following:

15 $$ \begin{aligned} & {\text{y}}(X_{R}^{1} ) = T_{1} {\text{y}}(X_{L}^{1} ) = {\text{y}}(X_{L}^{2} ),{\text{y}}(X_{R}^{2} ) = T_{2} {\text{y}}(X_{L}^{2} ) = T_{2} T_{1} {\text{y}}(X_{L}^{1} ) = {\text{y}}(X_{L}^{3} ),{\text{y}}(X_{R}^{3} ) = T_{3} {\text{y}}(X_{L}^{3} ) = T_{3} T_{1} T_{2} \\ & {\text{y}}(X_{L}^{1} ) = {\text{y}}(X_{L}^{4} ) \\ & {\text{y}}(X_{R}^{n} ) = T_{n} T_{(n - 1)} \ldots T_{1} {\text{y}}(X_{L}^{1} ) = T{\text{y}}(X_{L}^{1} ) \\ \end{aligned} $$

Ultimately, the displacement and stress at the left end of the first layer (X = ${X}_{L}^{j}$) in a unit cell are related to those at the right boundary of the nth layer (X = ${X}_{R}^{j}$) by the cumulative transfer matrix, $T.$

16 $$ T_{j} = \left[ {\begin{array}{*{20}c} {\cos \left( {K_{j} d_{j} } \right)} & {\frac{1}{{Z_{j} }}\sin \left( {K_{j} d_{j} } \right)} \\ { - Z_{j} \sin \left( {K_{j} d_{j} } \right)} & {\cos \left( {K_{j} d_{j} } \right)} \\ \end{array} } \right] $$

Here N is the number of unit cells and T is the accumulative transfer matrix. The transmission coefficient of the 1D PC structure is given in the form^63^:

17 $$\frac{{U}_{e}}{{U}_{0}}=\frac{{2E}_{0}({T}_{11}{T}_{22}-{T}_{12}{T}_{21})}{{E}_{0}\left({T}_{11}-{E}_{e}{T}_{21}\right)-({T}_{12}-{E}_{e}{T}_{22})}$$

where ${E}_{0}$ and ${E}_{e}$ are the Young’s moduli of the two semi-infinite solids at the left and right of the structure. ${U}_{0}$, ${U}_{e}$ are the incident and transmitted amplitudes, respectively. ${{\varvec{T}}}_{{\varvec{i}}{\varvec{j}}}=\mathrm{T}(\mathrm{i},\mathrm{j})$ represents the elements of the total transfer matrix.

18 $${T}_{j}(1,1) = {T}_{j}(2,2)= cos({K}_{j}{d}_{j})$$

19 $${T}_{j}\left(\mathrm{1,2}\right)=\frac{1}{{Z}_{j}}\mathrm{sin}({K}_{j}{d}_{j})$$

20 $$ T_{j} \left( {2,1} \right) = Z_{j} \sin \left( {K_{j} d_{j} } \right) $$

where ${Z}_{j}=\frac{{E}_{j}\upomega }{{\mathrm{C}}_{\mathrm{L}}j}$, ${\mathrm{C}}_{\mathrm{L}}j$ is the longitudinal wave velocity in each layer, ${d}_{j}$ is the thickness of each layer of the unit cell.

## Methods

**Figure 2:**
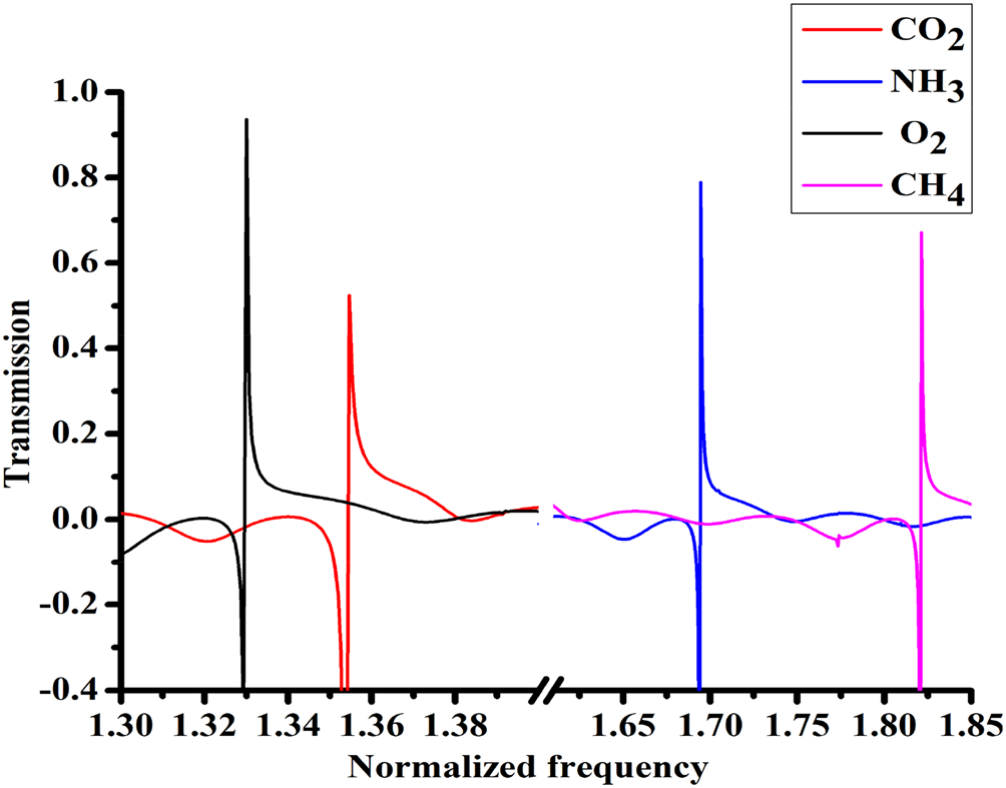
The transmission spectra of the 1D-DPC gas sensor structure as a function of the normalized frequency at room temperature.

Our proposed 1D-DPC structure has the ability to sense any type of gases with different sensitivity for each gas. Thus, when the acoustic wave normally is incident on the our 1D-DPC structure which consists of [(Lead/Epoxy)^2^ − (sensing material) − (Lead/Epoxy)^2^], each layer in the structure has mechanical properties different from each other layers. For instance, the speed of sound and density of lead are different from the epoxy and gas layer as given in Table 1. So, due to the periodicity of our structure as shown in Fig. 1, the mechanical properties of the structural layers such as sound speed and density are changing periodically. Wherefore, the incident acoustic wave disperses into several waves and the diffraction of these waves interferes with each other at each interface between layers of our proposed structure. If the interference was a constructive interference, it results in the formation of the stopped PC band gaps^64,65^. On the other side, when the interference is destructive, the resulted band is a transmission band^27,66^. The formula of the incident wave equation was written in Eq. (5) in the theoretical model part. In addition, Eq. (17) given the formula of the transmitted wave’s coefficient that describes the transmitted waves through the structure. Concerning the interaction between gases and PC, we supposed to insert a defect layer inside the perfect phononic structure. As a result, the interaction between any types of gases that inserted as a defected layer in 1D-PC, it can store some energy of the incident acoustic wave expressed in the formation of Fano-resonance peaks inside the transmitted band gaps as shown in Fig. 2. With changing in the type of gas, the Fano resonance peak frequency and intensity will change as well. This Fano resonance peaks enable our 1D-DPC gas sensor to detect efficiently the type and physical properties of the target gases with high sensitivity, Q-factor and figure of merit. It is seen that the 1D-DPC gas sensor has sensitivity values of 292, 207, 202, and 103 MHz/(kg/m^3^) for O_2_, NH_3_, CO_2_, and CH_4_ gases, respectively. Moreover, our proposed 1D-DPC can be implemented experimentally and theoretically easily due to the use of 1D multilayered structures in sensing applications that were introduced in several works of literature^43,52–55^. This work is a full theoretical and simulation investigation. However, the proposed design can easily be experimentally fabricated similar to identical 1D-PCs structures such as those listed in the past articles^26,54^. Also, our study of the 1D-PC as a gas sensor based on the TMM through MATLAB program as described in detail in the theoretical model.

## Results and discussion

### 1D-DPC high sensitivity gas sensor

In this work, to the best of our knowledge, it is the first time that an ultrahigh sensitive gas sensor towards CO_2_, CH_4_, NH_3_, and O_2_ gases based on the TMM and Fano resonance of a 1D-PC structure is proposed. Basically, our novelty is focused on investigating a novel gas sensor based on the 1D-PC which covered many sides that do not cover yet as introduced in all previous work of 1D or 2D PCs gas sensors^1,39–42^.

We introduce the output simulated results based on the mentioned mathematical model. The structure is designed as shown in Fig. 1 with incident acoustic wave of angle = 0°. We used lead and epoxy as consistent materials due to the large acoustic impedance mismatch between them. The idea of gas sensing is studied experimentally by 2D Surface PC Ring Resonators^1^. Also, the interaction mechanisms between the gaseous and acoustic waves are presented^40^. Moreover, the acoustic properties of a hole in a wall of finite thickness are experimentally introduced before^26,67^. However, the field of studying 1D-DPC as a toxic gas sensor based on the TMM and Fano resonance still not covered yet, which is the main focus of this study. The properties of materials used in calculations are given in Table 1. Here, as shown in Fig. 2, we present the transmission spectra of the binary 1D-DPC gas sensor is plotted versus the normalized frequency ($\omega$a/2π $c$), where $c$ is the sound speed in epoxy of 1300 m/s.

Firstly, the Fano resonance transmission spectrum gives the selectivity of the 1D-DPC gas sensor towards O_2_, CO_2_, NH_3_, and CH_4_ gases as seen in Fig. 2. Obviously an appearance of sharp Fano resonance transmitted peaks for O_2_, CO_2_, NH_3_, and CH_4_ gases. Each Fano resonance peak refers to the acoustic properties of each gas. For the case of O_2_, a higher sharp Fano resonance transmitted peak than the other gases with intensity about 94% at a normalized frequency of 1.33. Meanwhile, the CO_2_, NH_3_, and CH_4_ gases have transmitted peaks with intensities about 52%, 79%, and 67% appeared at normalized frequencies of 1.35, 1.69, and 1.82, respectively. Due to the lower acoustic speed of sound in O_2_ and CO_2_ than in NH_3_, and CH_4_, it has seen that the transmitted peaks of NH_3_ and CH_4_ gases are shifted to higher frequencies range than O_2_, and CO_2_.

Secondly, we have studied the Fano resonance peaks for each gas. Fano resonance is resulted from the destructive interference between a discrete quantum state and a continuum band of states, and occur at the interface between the lead, epoxy, and the gas layer^17,33,67^. The novelty of sharp line shape of Fano resonance peaks indicating a significant effect on the sensitivity measurements. Usually, the presence of Fano resonance in any sensor design enhances the sensitivity and quality factor values, this is the major purpose of introducing Fano peak s in our sensor design. The sharper of asymmetric line shape of the Fano resonance can be directly effect on the observed high sensitivity value toward different gases^51^. As observed in Fig. 2 we have very sharp and asymmetric resonant profiles that used to detect and compare several important gases such as O_2_, CO_2_, NH_3_, and CH_4_. Also, they demonstrated a high performance gas sensor based 1D-DPC.

Further, we have been observed sharp Fano resonance peaks which mainly contributed to the high sensitivity records of the proposed gas sensor structure in the MHz frequency range. Also, as shown in Fig. 2, with increasing the acoustic speed of sound of O_2_, CO_2_, NH_3_ and CH_4_ gases, the Fano resonance peaks shifted toward higher frequencies. In addition, there was a loss in Fano resonance transmission peaks (negative transmission). This is owing to that the acoustic energy is extracted to the gas defect layer from a region (lead and epoxy layers) much larger than the physical dimension of the gas defect layer itself^67^. Also, when the acoustic wave interacts with our optimized structure, the gas defect layer considered as empty hole. The negative transmission value indicated a transmission coefficient larger than unity; this implies that the acoustic energy transmitted through the gas defect layer exceeds that incident upon the hole.

### Sensor analysis

The performance and efficiency of any sensor are determined by many parameters such as the sensitivity (S), quality factor (Q), and figure of merit (FOM). These parameters can be obtained by using the following expressions^68–70^:

21 $$S=\varDelta {f}_{r}/\varDelta x$$

22 $$Q={f}_{r}/FWHM$$

23 $$FOM=S/FWHM$$

where ${f}_{r}$ is the resonance frequency, ${f}_{r(air)}=1.25\times {10}^{9}$ Hz of the air was used as a reference to calculate $\varDelta {f}_{r}$ as: ${\varDelta f}_{r}={f}_{r}\left(gas\right)-{f}_{r}\left(air\right)$, ∆x change of input parameter (density or temperature), and FWHM is the full width at half maximum of the resonant peak.

**Figure 3:**
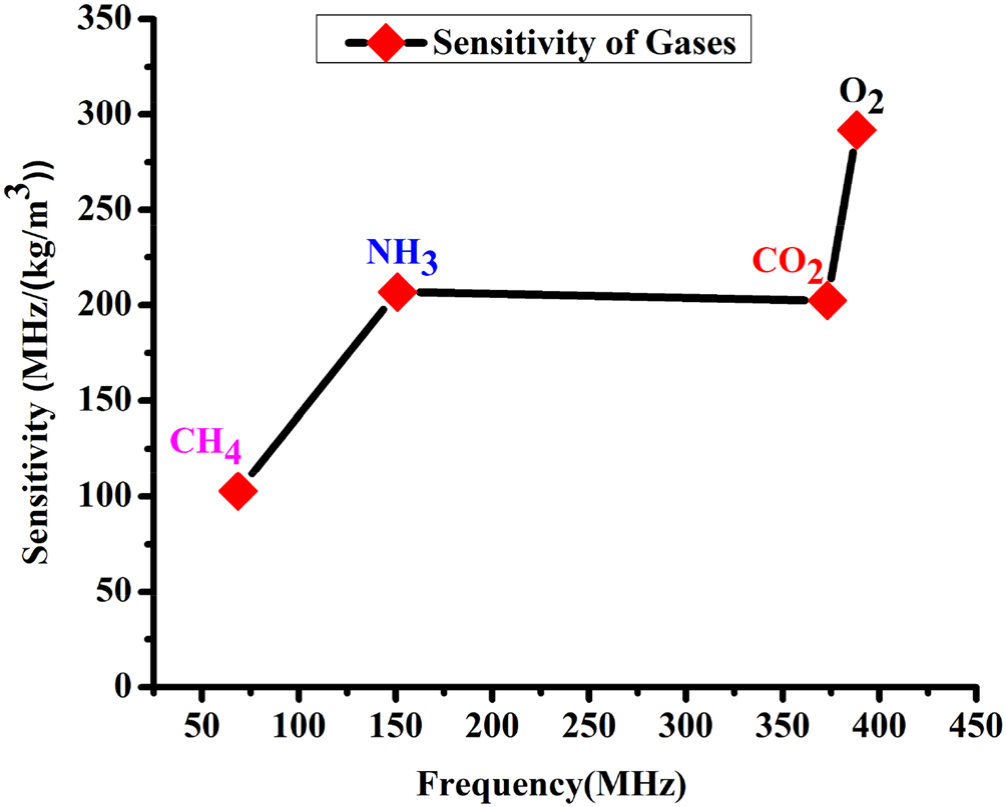
The sensitivity of the [(Lead/Epoxy)^2^ − (different gases) − (Lead/Epoxy)^2^] 1D-DPC gas sensor structure at different target gases as a function of the frequency.

Figure 3 illustrates the sensitivity of the1D-DPC sensor towards O_2_, CO_2_, NH_3_, and CH_4_ gases at room temperature (RT = 20 °C) is plotted with frequency. Using Eq. (21), S is calculated at the peak of Fano resonance for our 1D-DPC gas sensor structure and the results are depicted in Table 2 and Fig. 3. The 1D-DPCs sensor indicates the sensitivity of O_2_, CO_2_, NH_3_, and CH_4_ gases. Sensitivity values of 292, 207, 202, and 103 MHz/(kg/m^3^) at frequencies values equal 388, 151, 373, and 68.6 MHz for O_2_, NH_3,_ CO_2_, and CH_4_ gases respectively. The sensor recorded a high sensitivity towards O_2,_ as it has the highest value of frequency followed by CO_2_, NH_3_, and CH_4_ gases. On the other hand, the sensor shows the lowest sensitivity towards CH_4_. Also, we have observed that, from Eq. (21), the sensitivity of gases depends on the frequency, due to the fact that with increasing the frequency, the sensitivity can be increased as well. Our proposed 1D-DPC sensor based on Fano resonance shows higher sensitivity towards CO_2_ gas than previously reported gas sensors of 17.64 MHz/ppm^39^. These sensors include a CO_2_ gas sensor based on the evanescent coupling of the spoof surface described by Cicek et al.^39^.

**Table 2:** Gives the values of ${S, f}_{r}, FWHM, FOM,$ transmission intensity, and Q at room temperature for the O_2_, CO_2_, NH_3_, and CH_4_ gases.

Gases	${{{f}}}_{{{r}}}$ (MHz)	Transmitted peaks intensity (%)	FWHM (MHz)	S (MHz/(kg/m^3^))	FOM (m^3^/Kg)	Q
O_2_	864	94	0.451	292	647	1920
CO_2_	880	52	0.840	202	241	1050
NH_3_	1100	79	0.508	207	418	2170
CH_4_	1180	67	0.390	103	263	3040

In order to design a high-performance gas sensor, the Fano resonance peaks should be sharp and with a high-quality factor. The high-quality factor values refer to an accurate sensor measurement. The S, FOM, FWHM, and Q are calculated at the peak of Fano resonance for each gas through the our1D-DPC gas sensor and reported in Table 2. These parameters were calculated based on Eqs. (21)–(23), the intensity of the transmitted peaks for O_2_, CO_2_, NH_3_, and CH_4_ gases. From Table 2, the sensitivity for O_2_, CO_2_, NH_3_, and CH_4_ gases are about 292, 207, 202, and 103 MHz/(kg/m^3^), respectively. It is showing a high sensitivity, Q, and FOM values. The Q and FOM values determine the efficiency and performance of the sensors. For CH_4_, the highest Q value of 3040 was observed, followed by NH_3_, O_2_, and CO_2_ of about 2170, 1920, and 1050, respectively. On the other side, the O_2_ has the highest value of FOM of about 647, followed by NH_3_, CH_4_, and CO_2_ of about 418, 263, and 241 m^3^/Kg, respectively. The CH_4_ gas has the highest Q value due to it has the lowest FWHM among the other gases, but the CO_2_ has the lowest Q because of its FWHM has the higher value. Hence the values of FWHM of the listed gases are as $FWHM_{{CO_{2} }}  > FWHM_{{NH_{3} }} > FWHM_{{O_{2} }}  > FWHM_{{CH_{4} }}$ as seen in Table 2. The observed high values of Q and FOM of the tested gases are due to the small broadening in the Fano resonance peaks of each.

### Effect of temperature on fano resonance position of 1D-DPC gas sensor

**Figure 4:**
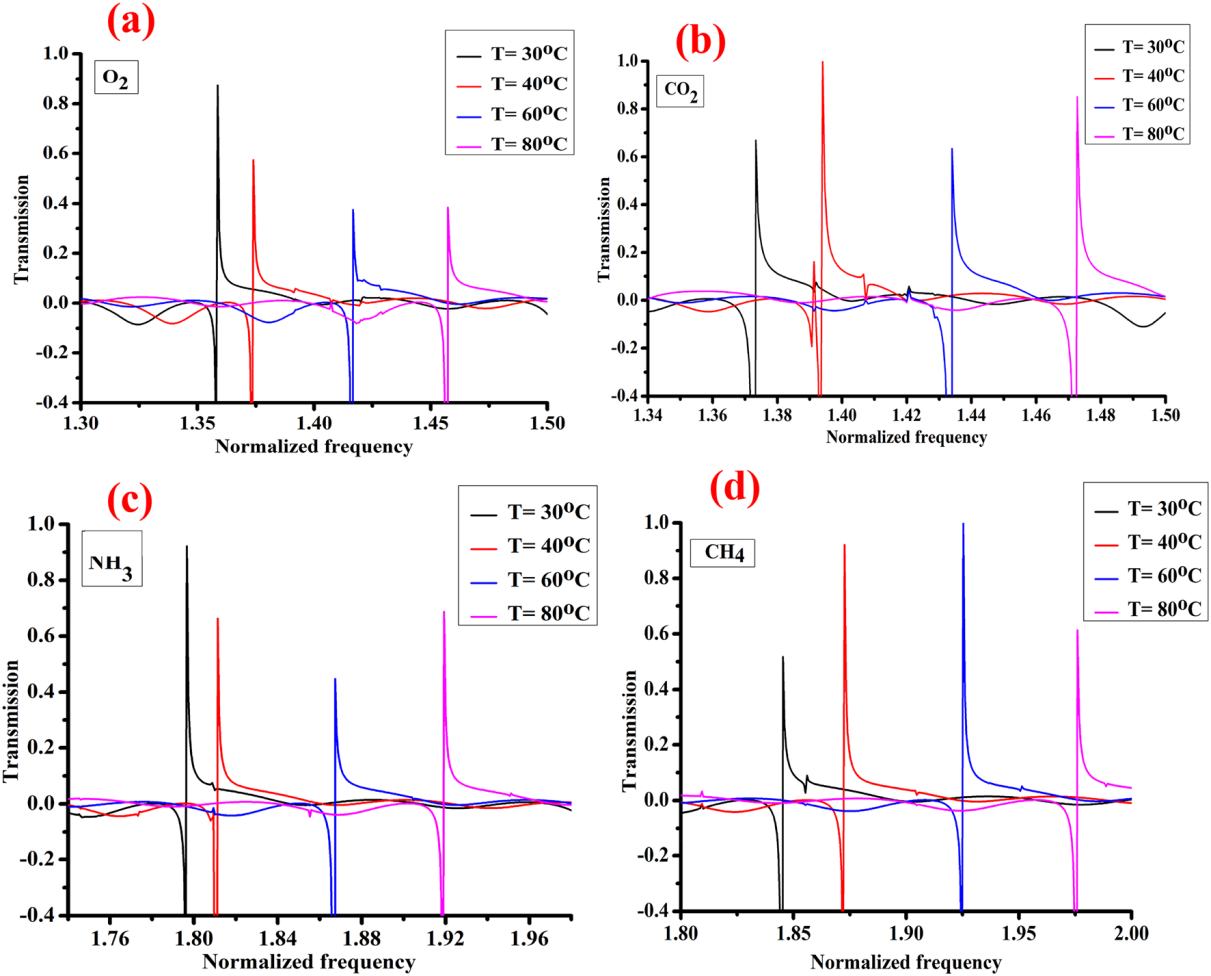
Shows the effects of temperature on the Fano resonance transmitted peak of the [(Lead/Epoxy)^2^ − (different gases) − (Lead/Epoxy)^2^] 1D-DPC gas sensor with a thickness of the gas defect layer of 1.5 μm. Where (**a**), (**b**), (**c**), and (**d**) representing O_2_, CO_2_, NH_3_, and CH_4_ gases.

In this section, we propose that the defect layer was filled with O_2_, CO_2_, NH_3_, and CH_4_ gases separately. As shown in Fig. 4a–d, we study the effects of temperature on the Fano resonance transmitted peaks of O_2_, CO_2_, NH_3_, and CH_4_ gases through the 1D-DPC gas sensor, the propagation of acoustic waves through a gas defect at different temperatures from 30 to 80 °C. Also, we studied the effect of temperature on the acoustic properties of gases which also effect on the Fano resonance transmitted peaks of each gas through the 1D-DPC gas sensor. It is known that the temperature has a direct effect on the density and the acoustic speed of gases whereas, with increasing the temperature, the acoustic speed of gases increased due to their density decreased^71,72^. Therefore, the position of the Fano resonance transmitted peaks for each gas shifted towards the high-frequency range^73^.

### Effect of temperature on the sensitivity and the quality factor of the 1D-DPC gas sensor

**Figure 5:**
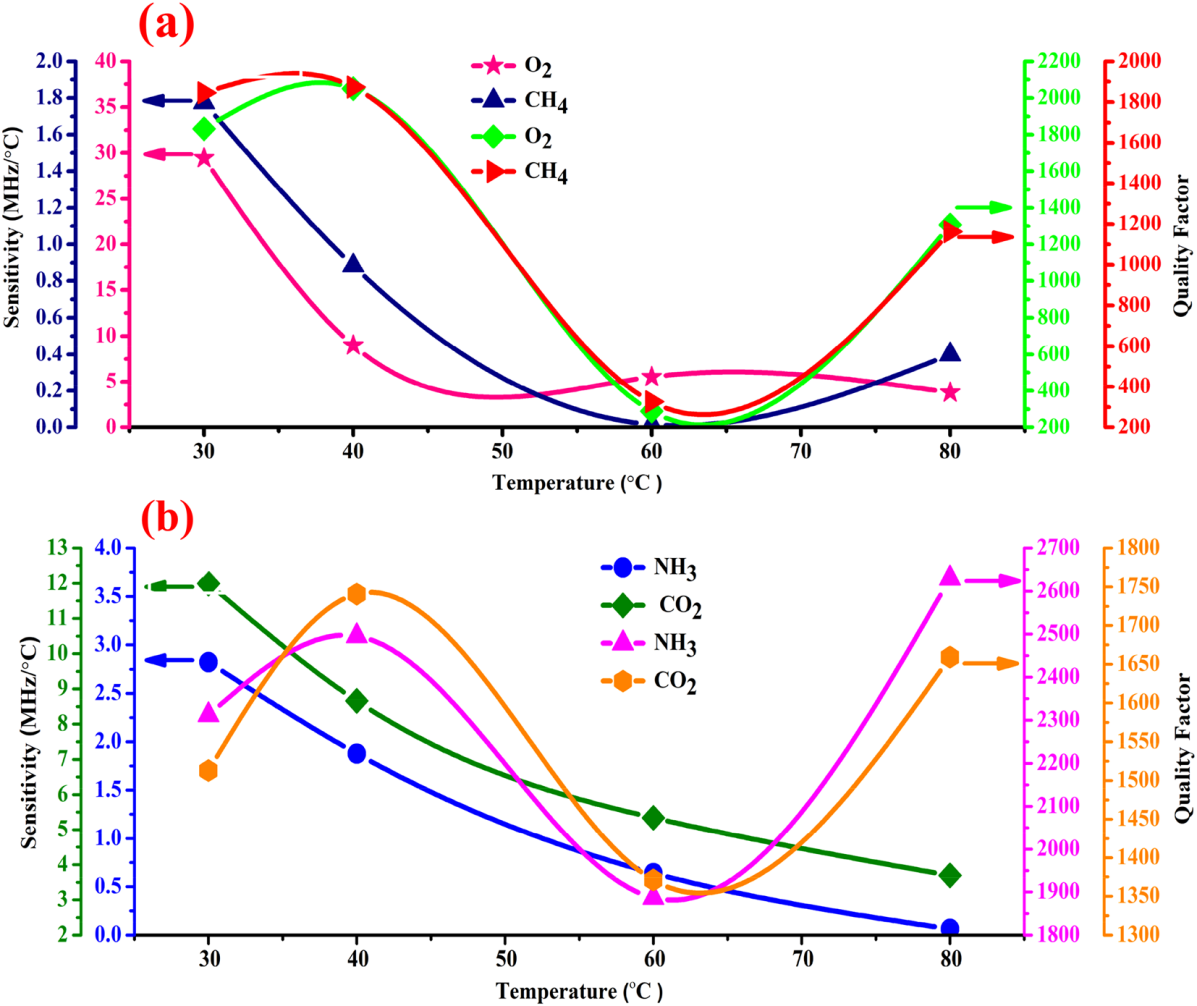
The effects of temperature on the sensitivity and quality factor of the [(Lead/Epoxy)2 − (different gases) − (Lead/Epoxy)2] 1D-DPC gas sensor. (**a**) O_2_–CH_4_ and (**b**) CO_2_–NH_3_ gases.

Actually, the temperature has a significant effect on the performance of the 1D-DPC gas sensor. As it affects the detection accuracy, which is defined as the sensor's ability to determine the resonance frequency of the sensing medium^28^. It seems that with increasing the temperature, the sensitivity decreased as shown in Fig. 5a,b and Eq. (21). An increase in the temperature can increase the acoustic speed of gases due to the decrease density value^72^. Therefore, ∆${F}_{r}$ decreased as given in Table 3, and the sensitivity is directly proportional to ∆${f}_{r}$ as indicated in Eq. (21). In Fig. 5a the sensitivity of 1D-DPC gas sensor for the O_2_ and CH_4_ gases have values of 29.4, 8.98, 5.53 and 3.82 (MHz/°C) and 1.77, 0.883, 0.0175 and 0.399 (MHz/°C) at 30, 40, 60, 80 °C, respectively. Also, as shown in Fig. 5a it’s observed that at 60 °C the sensitivity value reaches 0.0175 (MHz/°C) while at 80 °C increased to 0.399 (MHz/°C). Due to the ∆${f}_{r}$ at 80 °C has value higher than 60 °C as shown in Table 3. Also, For CO_2_ and NH_3_ gases as seen in Fig. 5b the values of sensitivity of these gases are 11.99, 8.65, 5.3 3 and 3.69 (MHz/°C) and 2.8, 1.87, 0.72 and 0.07 (MHz/°C) at 30, 40, 60, 80 °C, respectively. Further, Fig. 5a,b present the change of the quality factor of the 1D-DPC gas sensor towards O_2_ and CH_4_ and CO_2_ and NH_3_. Figure 5a shows the change of the quality factor of O_2_ and CH_4_ with temperature. It is well-known that the quality factor is a measure of the sharpness of the resonance peak, the larger the quality factor, the sharper the peak^74^. It is seen that the quality factor of CH_4_ has the highest values at 30 and 40 °C which equals 1845 and 1873, respectively, followed by O_2_ gas with 1833 and 2051 at 30 and 40 °C, respectively. This is because that the FWHM of O_2_ and CH_4_ have the lowest values of (0.48 and 0.44) and (0.65 and 0.65) MHz for 30 °C and 40 °C, respectively, as shown in Fig. 8a.

**Table 3:** The resonance frequency values with the corresponding physical properties of CH_4,_ NH_3_, CO_2_, and O_2_ gases at different temperatures.

Gases	Temperature (°C)	Density (kg/m^3^)	Longitudinal sound speed (m/s)	Resonance frequency (GHz)	∆${f}_{r}$ (Hz)
CH_4_	30	0.673	452	1.2	$5.32\times {10}^{7}$
	40	0.617	459	1.217	$3.53\times {10}^{7}$
	60	0.58	471.4	1.251	$1.05\times {10}^{6}$
	80	0.547	484	1.284	$3.19\times {10}^{7}$
NH_3_	30	0.6822	440	1.1678	$8.47\times {10}^{7}$
	40	0.6593	443.6	1.177	$7.51\times {10}^{7}$
	60	0.619	457.1	1.214	$3.84\times {10}^{7}$
	80	0.5832	469.9	1.247	$5\times {10}^{6}$
CO_2_	30	1.7777	270.7	0.89	$3.60\times {10}^{8}$
	40	1.7201	274.7	0.91	$3.46\times {10}^{8}$
	60	1.6155	282.6	0.93	$3.20\times {10}^{8}$
	80	1.523	290.2	0.96	$2.95\times {10}^{8}$
O_2_	30	1.27	333	0.88	$3.69\times {10}^{8}$
	40	1.229	336.7	0.89	$3.59\times {10}^{8}$
	60	1.155	347.1	0.92	$3.32\times {10}^{8}$
	80	1.089	357	0.947	$3.05\times {10}^{8}$

On the other hand, as shown in Fig. 5a at 60 °C and 80 °C the quality factor decreased as the FWHM increased, which have low values (289 and 1307) and (327 and 1163) at high values of FWHM (3.19 and 0.73) and (3.83 and 1.11) MHz for O_2_ and CH_4_, respectively, as shown in Fig. 8a. As well as, in Fig. 5b we have seen that the changing of the quality factor with temperature for CO_2_ and NH_3_ gases.

The quality factor has high values at 40 °C and 80 °C equals (1741 and 1660) and (2495 and 2630) respectively, thus as the CO_2_ and NH_3_ gases has the lowest values of FWHM which equals (0.52 and 0.57) and (0.471 and 0.474) MHz at 40 °C and 80 °C respectively. On the other side, at 30 °C and 60 °C the quality factor has lower values which equal (1513 and 1371) and (2312 and 1887) respectively. This due to CO_2_ and NH_3_ gases have high values of FWHM than at 40 and 80 °C, which equal (0.59 and 0.68) and (0.51 and 0.64) MHz, respectively.

### Relation between resonance frequency and the acoustic gas parameters of 1D-DPC gas sensor at different temperature

**Figure 6:**
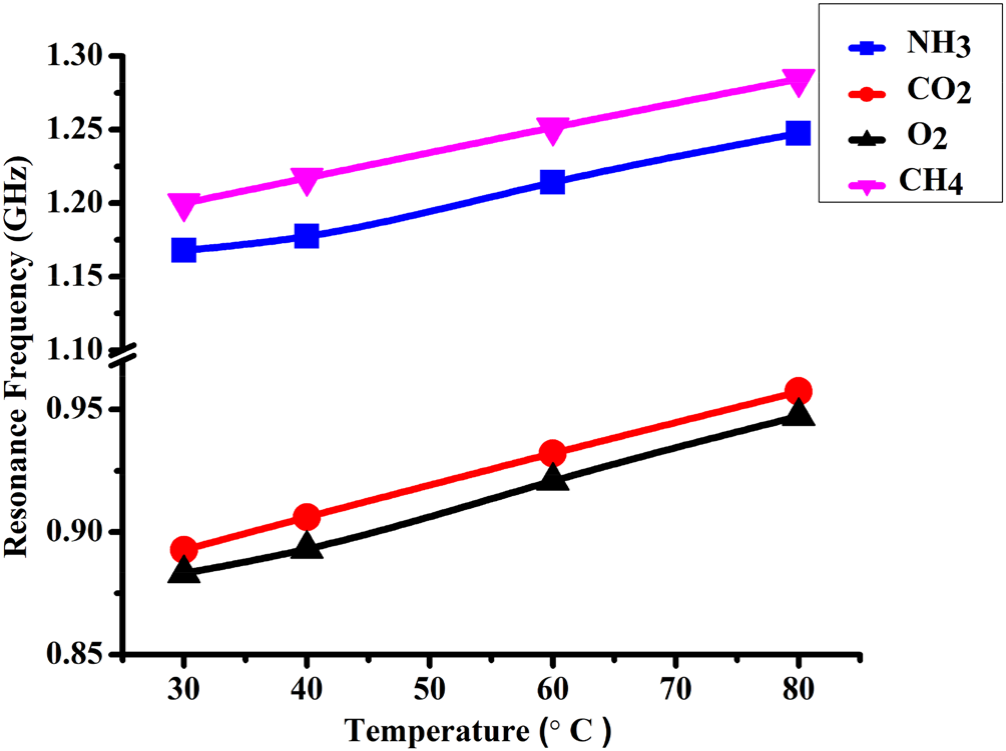
Shows the effect of temperature on the resonance frequency of [(Lead/Epoxy)^2^ − (different gases) − (Lead/Epoxy)^2^] 1D-DPC gas sensor towards NH_3_, CO_2_, O_2_, and CH_4_.

From Fig. 6 and Table 3, we related each resonance frequency value of these gases with its specific acoustic property. Table 3 gives the resonance frequency values with some acoustic properties of each gas at different temperatures. We have seen that with increasing the temperature, the density of each gas decreased and the acoustic speed of sound increased as well^71,72^. In Fig. 6, the resonance frequency increased with increasing the temperature for each gas. Moreover, CH_4_ gas has the highest resonance frequency value followed by NH_3_, CO_2_, and O_2_ gases, respectively due to the fact that CH_4_ has the lowest mass density and highest acoustic speed of sound among the other gases.

### The dependence of the sensitivity of the 1D-DPC gas sensor on the sound velocity at different temperatures towards NH_3_, CO_2_, O_2_, and CH_4_ Gases

**Figure 7:**
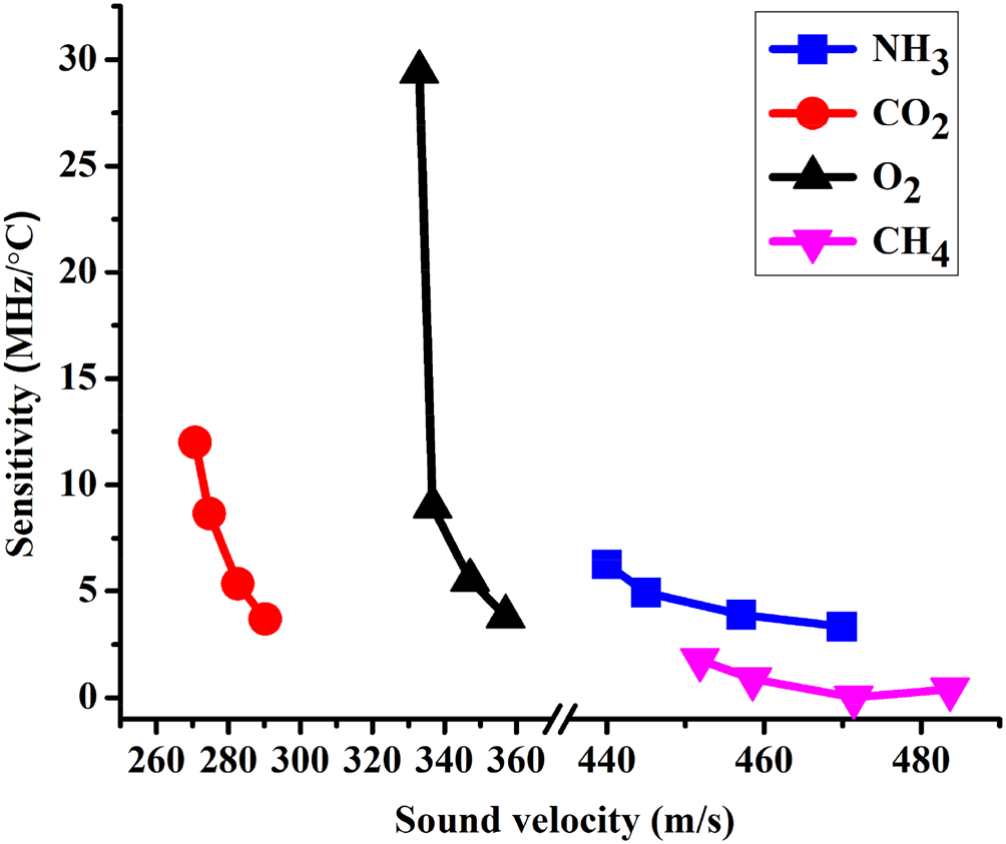
Shows the sensitivity of 1D-DPC gas sensor dependence on the sound velocity at different temperatures towards NH_3_, CO_2_, O_2_ and CH_4_ gases. All lines have four dots which are corresponding to temperature values, whereas the higher point value is referred to 30, followed by 40, 60, and 80 °C respectively.

The dependence of 1D-DPC gas sensor sensitivity on the sound velocity at different temperatures from 30 to 80 °C are showing in Fig. 7. From Fig. 7 we can see that with increasing the sound velocity as the temperature increased, the sensitivity of our proposed sensor towards CH_4,_ NH_3_, CO_2_, and O_2_ gases decreased. When the temperature increases, the sound velocity also increases due to the density of gas decrease as seen in Table 3. Also, ∆${F}_{r}$ decreased and the sensitivity is directly proportional to ∆${f}_{r}$ as indicated in Eq. (21) as given in Table 3. As presents in Fig. 7, our proposed 1D-DPC gas sensor abled to detect the lowest possible signal for CH_4_ which has the highest sound velocity with increasing the temperature. As the temperature increased from 30 to 80 °C, the speed of sound through each gas increased to higher values and the sensitivity of the sensor decreased as seen in Fig. 7.

### Effect of temperature on the FWHM and FOM of the 1D-DPC gas sensor towards NH_3_, CO_2_, O_2_, CH_4_ Gases

**Figure 8:**
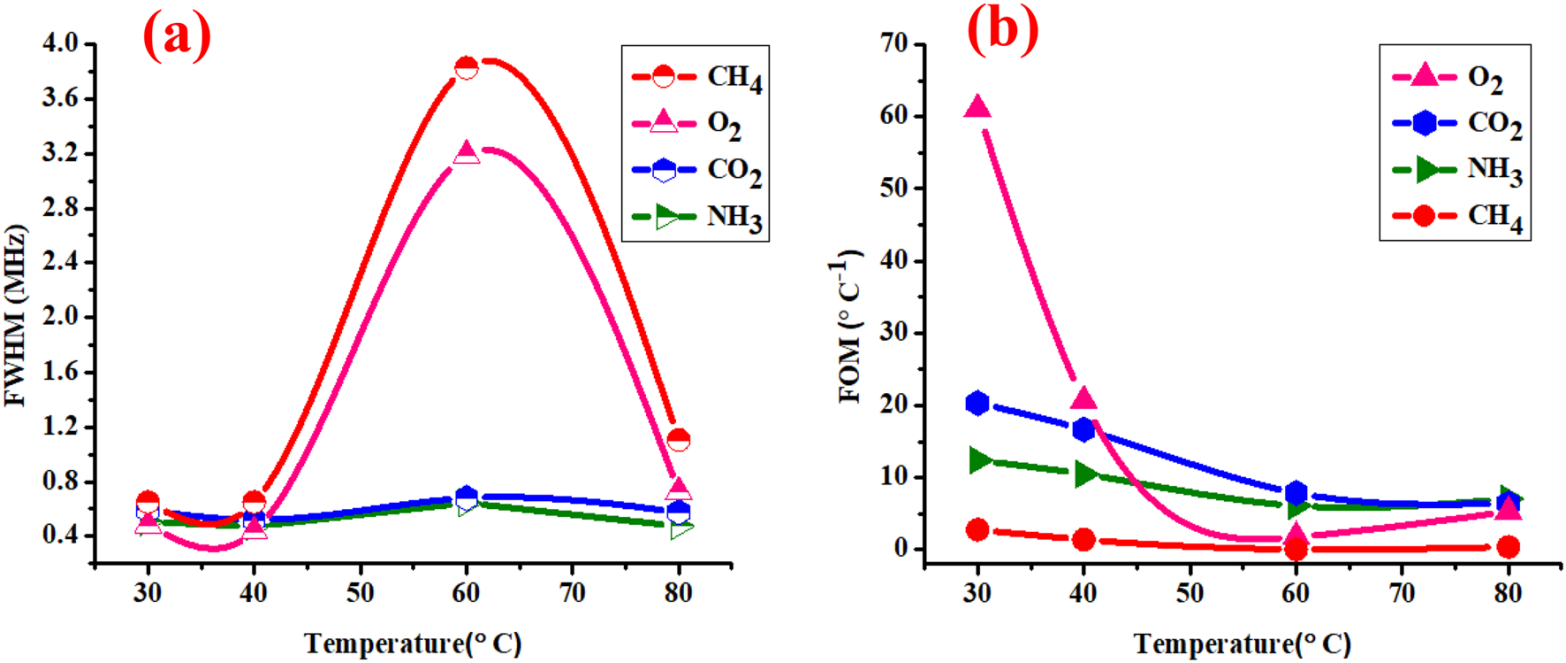
Shows the effect of the temperature on the FWHM and FOM of (**a**) NH_3_, (**b**) CO_2_, (**c**) O_2_ and (**d**) CH_4_ gases embedded in (lead / gas defect / epoxy) 1D-DPC gas sensor.

As presented in Fig. 8a,b, we studied the effect of temperature on the full width at half maximum (FWHM) of the Fano resonance transmitted peaks and the figure of merit (FOM) of the1D-DPC gas sensor towards NH_3_, CO_2_, O_2_, and CH_4_ gases. The detection accuracy is inversely proportional to FWHM of the Fano resonance transmission peak^75^. Our 1D-DPC gas sensor shows a high detection accuracy at a different temperatures which has a narrow resonance frequency peaks. The effect of temperature on the FWHM of transmitted peaks is shown in Fig. 8a. For CH_4_ and O_2_ gases, the FWHM has the highest values at 60 °C followed by 80 °C which equals (3.83 and 1.11) and (3.19 and 0.73) MHz, respectively. Meanwhile, the FWHM showed the lowest values (0.65 and 0.65) and (0.48 and 0.44) MHz at 30 and 40 °C, respectively. For the CO_2_ and NH_3_ gases at 30 and 60 °C the FWHM has values higher than at 40 and 80 °C, which equals (0.59 and 0.68), (0.51–0.64) MHz respectively as shown in Fig. 11a. This means that our gas sensor provides high-quality factors for CH_4_ and O_2_ gases at 30 and 40 °C. On the other side, for the CO_2_ and NH_3_ gases it shows high quality factor at 40 °C and 80 °C.

As observed in Fig. 8b, the temperature has an obvious effect on the FOM of the 1D-DPC gas sensor towards each gas. The FOM is the parameter indicates the efficiency of gas sensor to measure any change in the resonance frequency^49^. From Fig. 8b, we can see that the FOM of our proposed gas sensor decreased with increasing the temperature from 30 to 80 °C. Also, the FOM of 1D-DPC gas sensor has the highest values at 30 °C which equals 61.1, 20.33, 12.377 and 2.73 towards O_2_, CO_2_, NH_3_, and CH_4_ gases, respectively. Meanwhile, the CH_4_ has the lowest FOM between the other gases as shown. In addition, from Fig. 8b we can show that the FOM of NH_3_, CO_2_, O_2_, and CH_4_ gases has the highest values at 30 and 40 °C, but with increased the temperature to 60 and 80 °C the FOM for these gases has large decrease. Table 4 shows the most recent studies in comparison with our designed gas sensor sensors.

**Table 4:** A comparison between our design sensitivity with previous sensors.

Type of device	Sensing material	Sensor parameters
Kaya et al. represented numerically and experimentally gas sensor based on a 1D-PC^41^	Carbon dioxide CO_2_ level in air	The CO_2_ shows sharp transmission peak appeared at frequency value equal 58.49 kHz
Cicek et al. proposed acoustic ring resonator as a 2D-PC for a high sensitivity gas sensor^1^	Binary mixtures of dry air with CO_2_ and CH_4_ gases	The sensitivity of 2D-PC CO_2_ shows 3 MHz/ppm
Cicek et al. designed theoretically and experimentally ultrasonic gas sensor for CO_2_ based on evanescent coupling of spoof surface acoustic waves between two surface PCs containing trapezoidal grooves on rigid slabs^39^	CO_2_ in dry air	Sensitivity of CO_2_ shows 17.70 MHz/ppm (theoretically) and 16.20 MHz/ppm (experimentally) Sensitivity is equal − 17.64 MHz/ppm, − 18.52 MHz/ppm and − 17.99 MHz/ppm at T = 20 °C, 30 °C and 40 °C, respectively
A. Mehaney et al., demonstrated theoretically a CO_2_ gas sensor based on a 1D porous silicon PC sandwiched between two thin rubber layers^42^	CO_2_ pollutions in the surrounding air	Gas sensor of CO_2_ pollutions in the surrounding air is based on the change in the transmitted resonant mode intensity The effects of temperature and pressure on the resonant peak intensity are introduced as well When the temperature increases from 20 to 200 °C, the resonant peak intensity decreases from 0.559 to 0.443% With increasing the pressure, the resonant peak has higher a transmission intensity
This work, 1D-DPC gas sensor is a binary structure composed of two different layers of (Lead–Epoxy) repeated in N = 4 unit cells and inserted a defect layer in midi of the structure. The defect layer will be filled with different gases that will be tested separately	O_2_ CO_2_ NH_3_ CH_4_	Sensitivity at room temperature are 292, 202, 207 and 103 (MHz/(kg/m^3^)) for O_2_, CO_2_, NH_3_, and CH_4_, respectively Sensitivity at different temperatures for the O_2_ and CH_4_ gases have values of 29.4, 8.98, 5.53 and 3.82 (MHz/°C) and 1.77, 0.883, 0.0175 and 0.399 (MHz/°C) at 30, 40, 60, 80 °C, respectively. For CO_2_ and NH_3_ gases as seen in Fig. 5b. The values of sensitivity of these gases are 11.99, 8.65, 5.3 3 and 3.69 (MHz/°C) and 2.8, 1.87, 0.72 and 0.07 (MHz/°C) at 30, 40, 60, and 80 °C, respectively

### Effect of the damping rate on 1D-DPC gas sensor

The damping rate describes how acoustic waves in the 1D-PC decays after a disturbance through the structure. Sometimes the damping can cause some loses and makes the oscillations to gradually decay in amplitude up to zero or attenuate. The experiments indicate that low-density materials can provide high damping of structural vibration if the wave speed in the material is sufficiently low. The TMM is prepared for studying the absence or presence of wave dissipation inside 1D-PC^59^, the distinction is made in the definition of ${Z}_{j}$ and ${K}_{j}$ as in Eq. (24):

24 $${Z}_{j}={K}_{j}({E}_{j}+{\eta }_{j}\lambda )$$

In the un-damped wave propagation, the relationship between the wavenumber and the frequency followed by ${K}_{j}$ = $\frac{{w}_{j}}{{\mathrm{C}}_{\mathrm{L}}j}$. But in the case of the damped wave, the relationship between the wavenumber and the damped wave frequency for layer *j* can be introduced as Eq. (25)^59^:

25 $$\frac{{q}^{2}{\left[ {\mathrm{C}}_{\mathrm{L}}j\right]}^{4}}{4}{\left[{K}_{j}\right]}^{4}-{\left[ {\mathrm{C}}_{\mathrm{L}}j\right]}^{2}{\left[{K}_{j}\right]}^{2}+{w}_{d}^{2}=0$$

where ${\mathrm{C}}_{\mathrm{L}}j=\sqrt{ {E}_{j}/{\rho }_{j}} ,$ $w={K}_{j }{\mathrm{C}}_{\mathrm{L}}j$. We can formulate two complex-conjugate solutions for ${\left[{K}_{j}\right]}^{2}$, from which we develop the explicit relation Eq. (26):

26 $${K}_{j }=\pm \frac{1}{q{\mathrm{C}}_{\mathrm{L}}j}\sqrt{2(1\pm \sqrt{1-{q}^{2}{w}_{d}^{2}}})$$

This equation is only valid if q has a non-zero value. The damping rate is related to the quality factor (Q) of 1D-PC gas sensor. Q describes the behavior of an oscillator or wave under damping condition, the higher Q indicates a lower rate of acoustic energy loss relative to the stored energy of the PC. We can calculate the damping rate as Eq. (27)^76^:

27 $$\zeta =\frac{1}{2*Q}$$

### Effect of damping rate on the FWHM of fano resonance transmitted peaks and the sensitivity of 1D-DPC gas sensor

**Figure 9:**
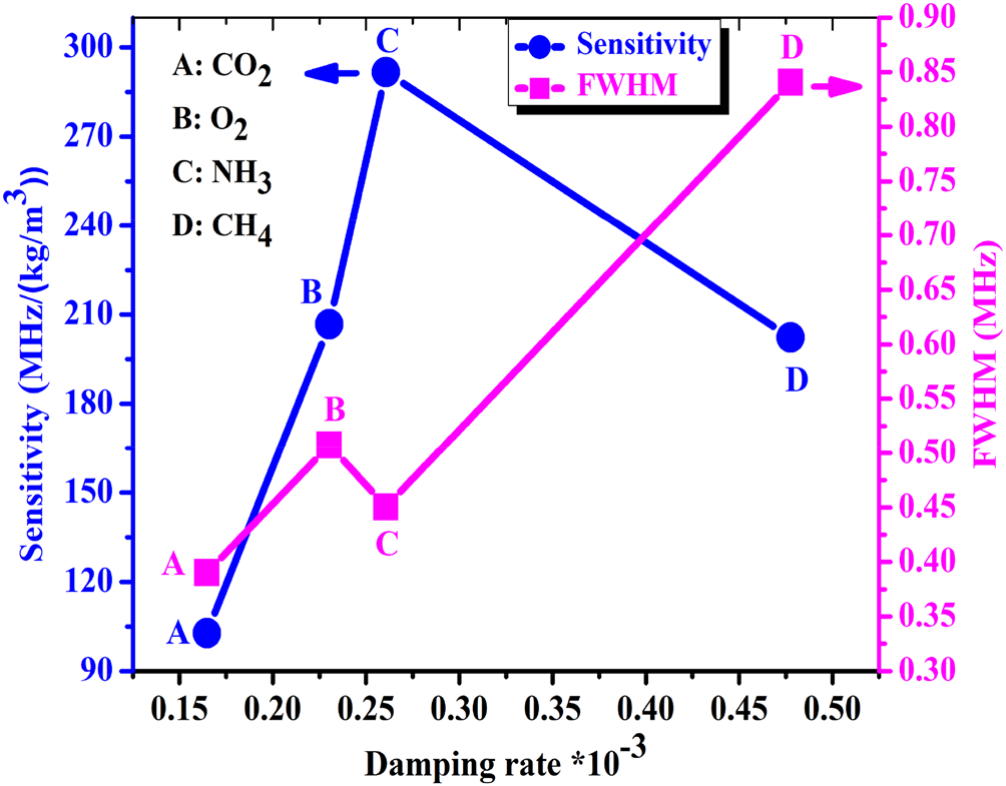
Shows the effects of the damping rate on the sensitivity and FWHM of 1D-DPC gas sensor towards (A): CO_2_, (B): O_2_, (C): NH_3_ and (D): CH_4_ gases.

As in Fig. 9, we studied the effect of damping rate on the full width at half maximum of the Fano resonance transmitted peaks (FWHM) and the sensitivity of the1D-DPC gas sensor towards NH_3_, CO_2_, O_2_, and CH_4_ gases at room temperature. We can see that the sensitivity increases from 103 to 292 (MHz/(kg/m^3^)) and the value of FWHM increases from 0.39 to 0.84 (MHz) with increasing the damping rate from $\zeta =0.16\times$ 10^–3^ to the value $\zeta =0.47\times$ 10^–3^ at room temperature as seen in Fig. 9.

**Figure 10:**
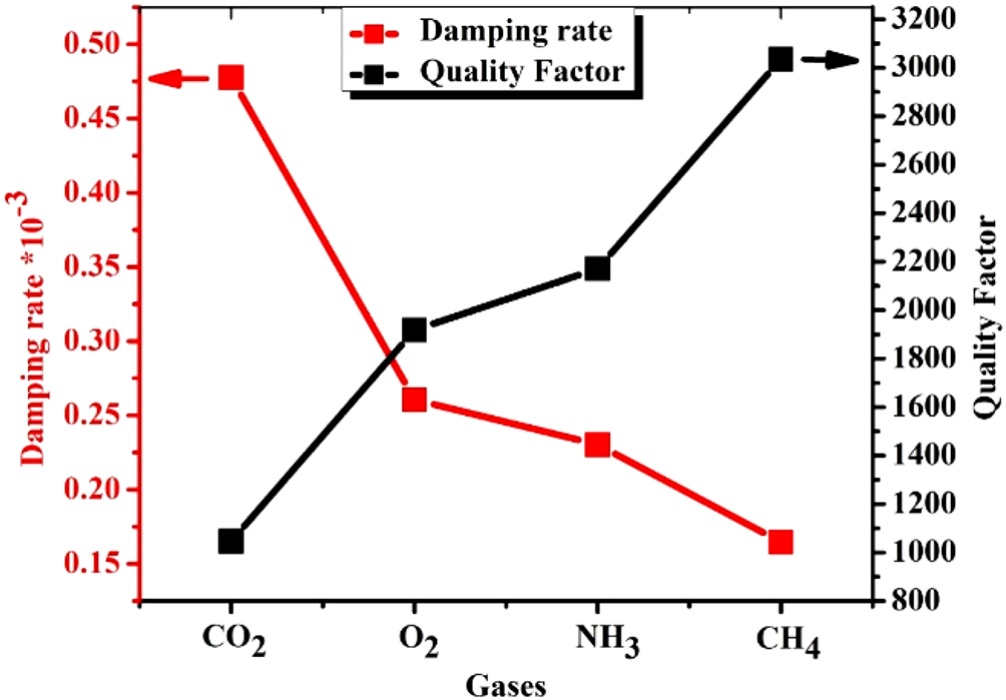
Shows the damping rate of acoustic wave inside 1D-PC gas sensor for different gases, and the relation between the quality factor and damping rate of 1D-PC gas sensor.

Figure 10 shows the damping rate of an acoustic wave through our optimum 1D-DPC gas sensor towards NH_3_, CO_2_, O_2_, and CH_4_ gases. We observed that the 1D-DPC has the highest value of the damping rate in CO_2_ and the lowest value for CH_4_ gas. The damping rate is inversely proportional to the quality factor of our gas sensor as seen in Eq. (27). The 1D-DPC gas sensor has the lowest quality factor value towards CO_2_ which equals 1050, but the CH_4_ has the highest value equals 3040 among other gases.

### Effect of temperature on the damping rate of 1D-DPC gas sensor

**Figure 11:**
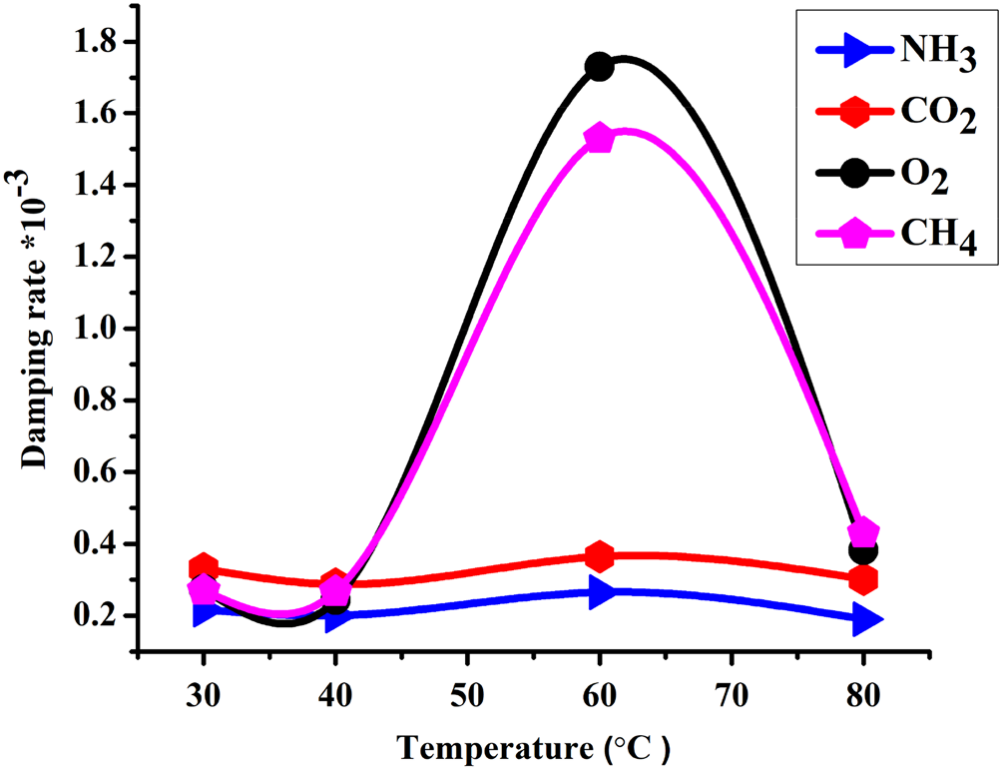
Represents the effect of the temperature on the damping rate of the acoustic wave inside 1D-DPC gas sensor forwards (**a**) NH_3_, (**b**) CO_2_, (**c**) O_2_ and (**d**) CH_4_ gases.

We studied the effect of temperature on the damping rate of acoustic wave inside 1D-PC gas sensor towards CO_2_, O_2_, NH_3_ and CH_4_ gases and plotted in Fig. 11. The designed 1D-DPC gas sensor shows a different damping rate at a different temperatures. For instance, CH_4_ and O_2_ gases, show the highest damping rate values at 60 °C followed by 80 °C which equals to (1.5 $\times$ 10^−3^ and 1.71 $\times$ 10^−3^) and (0.41 $\times$ 10^−3^ and 0.35 $\times$ 10^−3^), respectively. Meanwhile, the lowest damping rate for CH_4_ and O_2_ gases are presented at 30 °C and 40 °C with values of (0.27 $\times$ 10^−3^ and 0.273 $\times$ 10^−3^) and (0.267 $\times$ 10^−3^ and 0.243 $\times$ 10^−3^), respectively. While, CO_2_ and NH_3_ gases having the lowest damping rate values. At 30 °C and 60 °C, the damping rate of CO_2_ and NH_3_ gases has values higher than at 40 °C and 80 °C, equals to (0.33 $\times$ 10^−3^ and 0.22 $\times$ 10^−3^) and (0.36 $\times$ 10^−3^ and 0.27 $\times$ 10^−3^) respectively as shown in Fig. 11. Due to the inversely relation between the damping rate and the quality factor as seen in Eq. (27), this means that our gas sensor provides high quality factor measurements for CH_4_ and O_2_ gases at 30 °C and 40 °C. On the other hand, for the CO_2_ and NH_3_ gases it shows high quality factor at 40 °C and 80 °C.

## Conclusion

To the best of our knowledge, for the first time, a defected phononic crystal based high sensitive gas sensor. Our study shows a remarkable sensitivity towards O_2_, CO_2_, NH_3_, and CH_4_ gases based on [(Lead/Epoxy)^2^ − (different gases) − (Lead/Epoxy)^2^] structure for the first time. The inserted gases in the defected layer, make specific Fano resonant peaks through the phononic band gaps that related to the properties of each gas. Based on our literature review, no complete study showed a sensitivity, quality factor, and figure-of-merit investigations based on the transfer matrix method. For the first time, our proposed phononic crystal gas sensor shows high sensitivity values due to the appearance of Fano resonance peaks. Moreover, we introduced the effect of damping rate of the incident wave inside our gas sensor on the sensitivity and FWHM. The effect The effect of temperature (30–80 °C) on the sensitivity, $FWHM$ and quality factor, and the damping rate of the [(Lead/Epoxy)^2^ − (different gases) − (Lead/Epoxy)^2^] 1D-DPC gas sensor for O_2_, CH_4_, CO_2_, and NH_3_ gases was studied and calculated.
